# Geometrical tradeoffs in graphene-based deeply-scaled electrically reconfigurable metasurfaces

**DOI:** 10.1038/srep08834

**Published:** 2015-03-06

**Authors:** Sara Arezoomandan, Berardi Sensale-Rodriguez

**Affiliations:** 1 Department of Electrical and Computer Engineering, The University of Utah, Salt Lake City, Utah 84112, United States

## Abstract

In this work we study the terahertz light propagation through deeply-scaled graphene-based reconfigurable metasurfaces, i.e. metasurfaces with unit-cell dimensions much smaller than the terahertz wavelength. These metasurfaces are analyzed as phase modulators for constructing reconfigurable phase gradients along an optical interface for the purpose of beam shaping. Two types of deeply-scaled metacell geometries are analyzed and compared, which consist of: (i) multi split ring resonators, and (ii) multi spiral resonators. Two figures of merit, related to: (a) the loss and (b) the degree of reconfigurability achievable by such metamaterials -when applied in beam shaping applications-, are introduced and discussed. Simulations of these two types of deep-subwavelength geometries, when changing the metal coverage-fraction, show that there is an optimal coverage-fraction that gives the best tradeoff in terms of loss versus degree of reconfigurability. For both types of geometries the best tradeoff occurs when the area covered by the metallic region is around 40% of the metacell total area. From this point of view, reconfigurable deeply-scaled metamaterials can indeed provide a superior performance for beam shaping applications when compared to not deeply-scaled ones; however, counterintuitively, employing very highly-packed structures might not be beneficial for such applications.

Terahertz technology is a growing technological field, which in recent years has been finding multiple emerging applications in diverse areas including: medical imaging, biochemical sensing, security, wireless communications, and so on^1^. In this context, future compact low-cost terahertz systems, such as beam steerers for MIMO communications, tunable flat lenses for terahertz cameras, etc., will demand components capable of achieving active beam-shaping at some degree. Reconfigurable terahertz metamaterials^2^ were shown capable of modulating the phase of an arbitrary terahertz beam^3^; these metamaterial phase modulators can be employed to construct arbitrary phase gradients in an optical interface, which is of special interest for terahertz beam-shaping applications. In this regard, via independently biasing each metacell, thus spatially controlling the phase-shift, arbitrary phase gradients can be constructed^3^, which in turn can shape the reflected or transmitted beams in accordance with the recently proposed generalized laws of reflection and refraction (generalized Snell's law)^4^. Our work: (i) discusses the use of reconfigurable deep-subwavelength metasurfaces for constructing arbitrary phase gradients for beam shaping applications, (ii) introduces two figures of merit that are related to the performance of these metasurfaces in the context of beam shaping applications, and (iii) discusses the geometrical tradeoffs is designing such structures and identify the metal coverage-fraction as an important parameter in this regard.

When a phase gradient is placed in the interface between two media of refractive index *n_t_* and *n_i_*, Snell's law of transmission should be rephrased as the generalized law of reflection and refraction^4^:
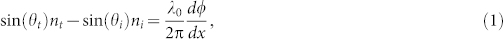
where *θ_i_* and *θ_t_* are the angle of incidence and the transmitted angle, respectively, *λ_0_* is the vacuum terahertz wavelength, and *dϕ*/*dx* represents the phase gradient. Assuming normal incidence and *n_i_ = n_t_* = 1, Eqn. (1) can be rewritten as:
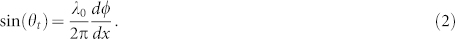
Therefore, it can be easily seen that the shape of the transmitted beam can be arbitrarily controlled via designing an adequate phase gradient. For instance, assuming an incident collimated beam, a linear phase gradient can tilt the transmitted beam, whereas a parabolic phase gradient can focus it. It is therefore of interest the use of electrically-driven reconfigurable metamaterial phase modulators for constructing these arbitrary phase gradients. However, in order to enable the design of these arbitrary phase gradients, each metacell in the device should be able to provide: (a) the same transmission amplitude, and (b) 360° (2π) control over the transmitted phase. In this context, it can be observed, as discussed by Chen et al^3^, that the terahertz transmission amplitude and phase through a metacell are not independent of each other, but they are related by Kramers-Kronig(KK) relations. Near frequencies where maximum amplitude modulation is achieved no phase modulation takes place. In contrast, near frequencies where the transmission amplitude is not dependent of the applied voltage (i.e. there is no amplitude modulation), but its slope is, the phase experiences maximum shift. From this point of view at the frequencies where maximum modulation of phase is obtained there is no amplitude modulation and therefore (a) is guaranteed. Since terahertz metamaterial phase modulators proposed to date exhibit phase modulation much smaller than 360° (see Refs. 3, 5), epitaxial stacking of multiple layers^3^ is necessary in order to achieve (b), which in turn increases the loss in the device. Moreover, construction of arbitrary phase gradients is also limited by the geometrical length of each unit-cell. This is due to the fact that, when employing metamaterials, a continuous phase gradient is approximated by a discretely spatially-varying one. From this point of view, the smaller the unit-cell length (when compared to the target terahertz wavelength), the better one can approximate an arbitrary phase gradient, therefore the more functionality and better performance the metamaterial beam shaper might achieve. In this context, a problem of metamaterial structures proposed to-date as phase modulators is that the unit-cell to wavelength ratio is not small enough to provide good performance. For instance, in order to provide ~90° control over the transmission angle using a 10 element phase-gradient discretization, a unit-cell length < *λ_0_*/10, thus a unit-cell to wavelength ratio < 0.1, is required. Terahertz metamaterial phase shifters reported to-date have unit-cell to wavelength ratios in the order of ~0.15/~0.2 (see Refs. 3, 5). From this point of view, *one of the main challenges of terahertz metamaterial phase modulators is: designing a metamaterial with small unit-cell to wavelength ratio, which has a large phase modulation and large transmission at the frequencies at which maximum phase modulation takes place*. In this work, the terahertz (THz) light propagation through deeply-scaled graphene-based reconfigurable metasurfaces is studied in the context of beam-shaping applications. Although graphene is used as an example reconfigurable semiconductor in these devices, the discussion presented here is general enough and the results are also valid if employing other semiconductor materials.

## Results

**Figure 1:**
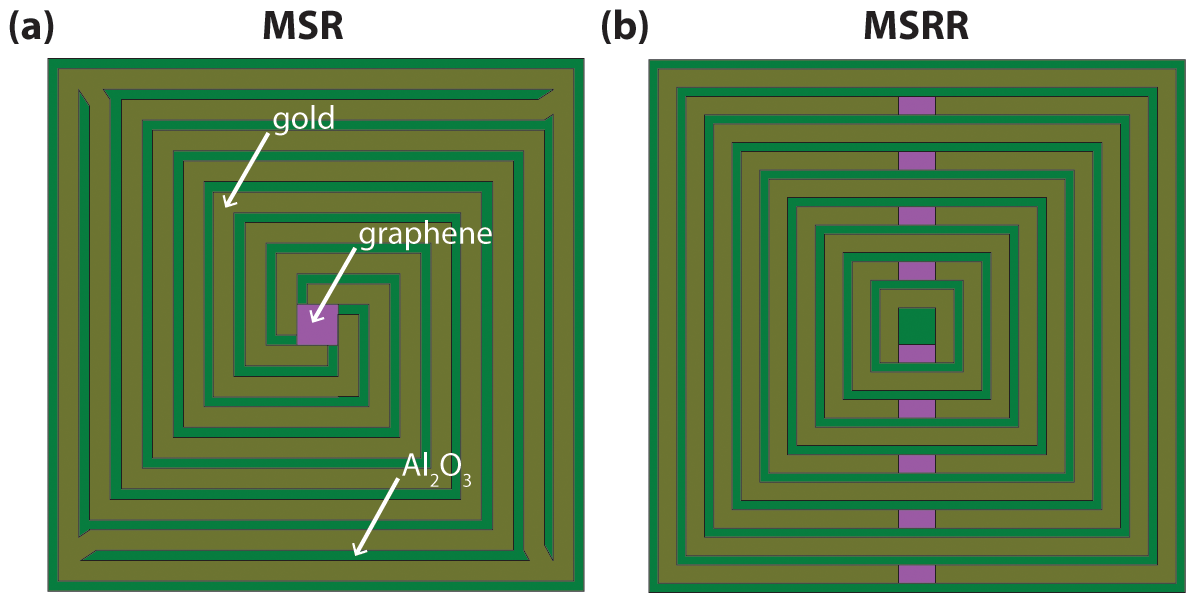
Sketches of the analyzed metacell geometries. (a) A multi spiral resonator (a), and (b) a multi split ring resonator.

Two types of deep-subwavelength metamaterial geometries are studied and compared. These consist of: (i) *multi spiral resonators* (MSRs), and (ii) *multi split ring resonators* (MSRRs), as depicted in Fig. 1(a) and Fig. 1(b), respectively. A sheet of graphene was considered as the tunable element to reconfigure the terahertz transmission properties of the metamaterial^6^,^7^,^8^, which was placed in some strategic regions of the device, as depicted in Fig. 1. For the MSRR structure the graphene sheet is located inside each split, whereas for the MSR structure the graphene sheet is located in the geometric-center of the structure connecting the four spiral arms. The electromagnetic properties of this graphene layer, and therefore the effective properties of the metamaterial, can be adjusted via controlling the Fermi level of graphene therefore its density of states available for intra-band transitions and thus its optical conductivity^6^. Although graphene metamaterials have been widely employed in devices modulating the amplitude of a transmitted terahertz beam^9^,^10^,^11^,^12^, to the author's knowledge, graphene-based terahertz metasurfaces controlling the phase of a transmitted terahertz beam under normal incidence, have not yet been proposed to-date. In terms of reflection, graphene based metamaterials have theoretically been shown capable of modulating phase in reflect-array geometries^13^; however these structures do not provide constant amplitude of reflection when the phase is reconfigured. Actuation over the graphene terahertz optical conductivity can be achieved electrostatically via either gating graphene with another graphene layer (self-gated structure)^14^, or via employing ion-gel as the gating element^15^.

**Figure 2:**
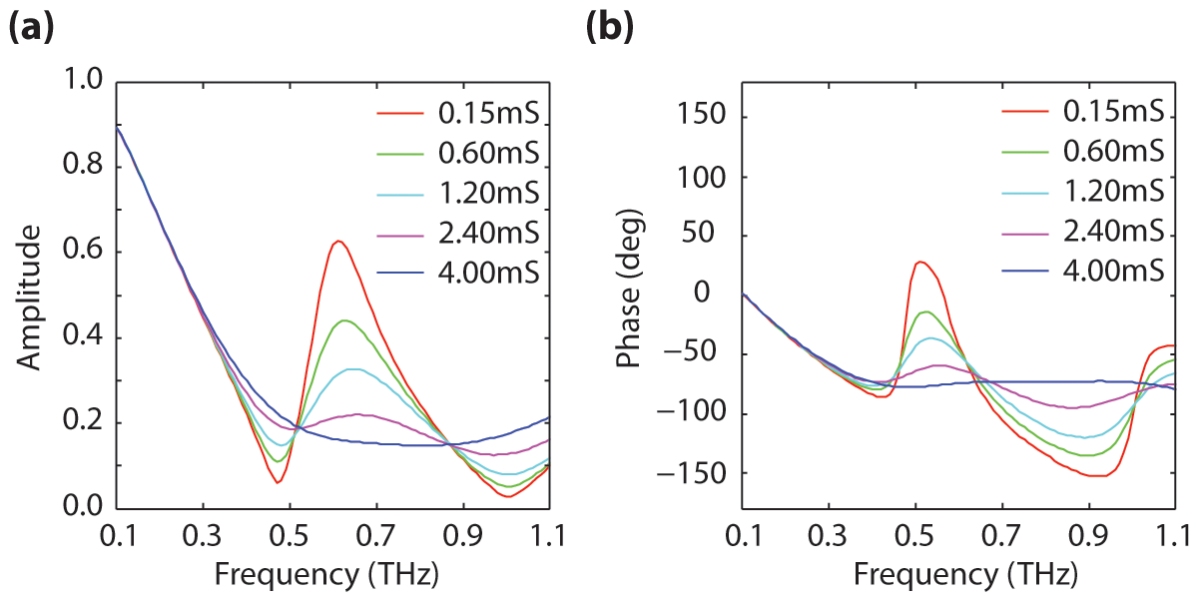
Characteristic response of one of the analyzed metamaterials. (a) Amplitude and (b) phase of the transmission as a function of frequency for different graphene conductivities -for a MSR with 30% metal coverage-fraction-. The conductivity of graphene is varied from 0.15 mS to 4 mS.

Shown in Fig. 2 are the characteristic transmission and phase frequency responses as a function of graphene conductivity for one of these metamaterials (a MSR with 30% metal to unit-cell area coverage-fraction). Maximum phase modulation, 108°, was observed at 500 GHz; at this frequency the transmittance was found to be 20%, independently of the graphene conductivity.

**Figure 3:**
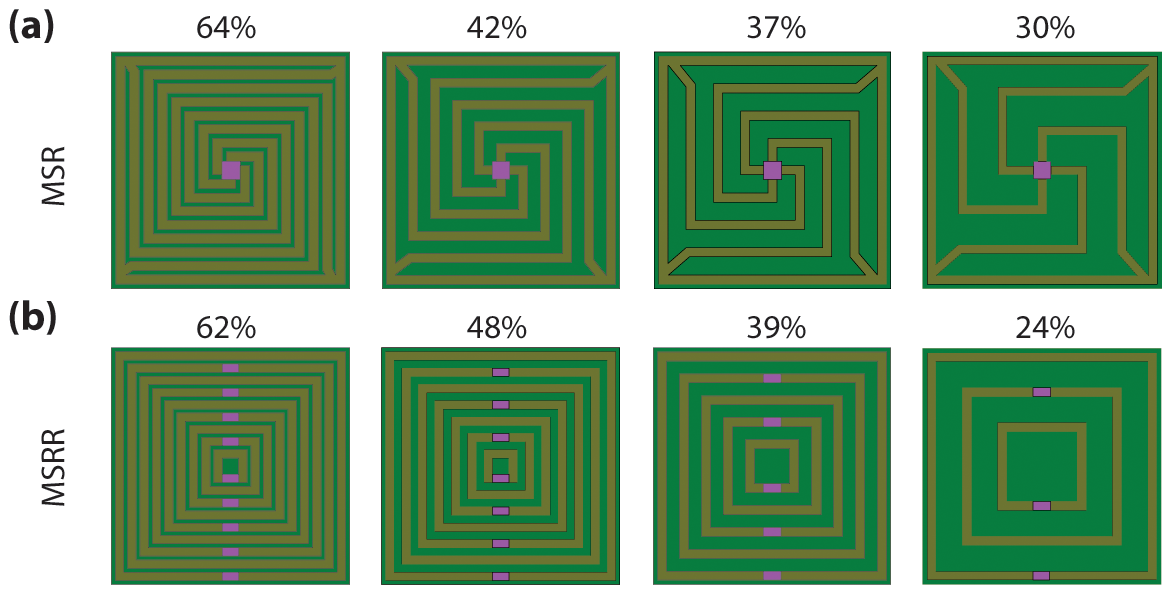
Sketch of the simulated metamaterial geometries. (a) Multi spiral resonators with metal coverage-fraction 64%, 42%, 37% and 30%, and (b) multi split ring resonators with metal coverage-fraction 62%, 48%, 39%, and 24%. The coverage-fraction is defined as the ratio between the area covered by metal and the total area of a metacell.

Metacells consisting of MSRRs and MSRs were numerically simulated. In order to extract useful information regarding the design tradeoffs in these structures, simulations were performed by changing the metal coverage-fraction in each of both geometries. The width of the metal rings/spirals was set to 2-μm, which is a dimension comparable with that of the minimum features achievable in optical lithography; the unit-cell edge-length was taken between 52-μm and 58-μm (depending on the particular metacell). The graphene sheet area was set to 4-μm by 4-μm in the MSR structure and 2-μm by 4-μm in the MSRR structure. Therefore, a larger coverage-fraction translates into: (a) a larger number of rings and smaller spacing between adjacent rings for the MSRR metacell geometries, or (b) a larger number of turns and smaller spacing in-between metals for the MSR metacell geometries. Shown in Fig. 3 are the sketches of the eight simulated devices (4 MSRR geometries and 4 MSR geometries, each of them having a different metal coverage-fraction); the results of these simulations are shown in Table 1 (where *f_p_* stands for the frequency at which maximum phase modulation takes place, and *PM* and *T* are the phase modulation and the transmittance, respectively, through the metacell at *f_p_*).

## Discussion

As discussed in the introductory section, for beam shaping applications, an ideal metamaterial geometry should provide: (i) large phase modulation, (ii) large transmittance, and (iii) small unit-cell to wavelength ratio. Arbitrary phase gradients need to be constructed when reconfiguring the phase-shift inserted by each metacell. Therefore, a full control of the transmitted phase, i.e. between 0 and 360°, is desirable in each unit-cell in order to achieve truly arbitrary designs. But the phase modulation achievable by each metacell is finite, e.g. prior metamaterial phase-modulator proposals^3^,^5^ show phase modulation < 50°; therefore, epitaxial stacking of layers is required in order to obtain a 360° control over phase in each metacell. When many layers are epitaxially stacked, although the phase shifts can be added^3^, loss increases with number of layers, which is not desirable. From this point of view, the following figure of merit, related to loss, is defined: *FoM_1_* = *PM* × *T*/([360°] × [100%]). For an ideal metacell geometry *FoM_1_* should approach unity (since *PM* and *T* are bounded by 360° and 100%, respectively); the larger the *FoM_1_* the most suitable a metamaterial geometry is for beam steering, i.e. the less loss the device will provide. But also, a small unit-cell to wavelength ratio is required in order to construct sharp phase gradients, which are needed, for instance, in order to achieve large swings in beam steering applications as discussed in the introductory section. From this point of view, a second figure of merit is defined: *FoM_2_* = *L*/*λ_P_*, where *L* is the edge-length of the metacell and *λ_p_* is the wavelength associated with the frequency at which maximum phase modulation takes place. For an ideal metacell geometry *FoM_2_* should approach zero, the smaller the *FoM_2_* the most suitable a metamaterial geometry is for beam steering.

**Figure 4:**
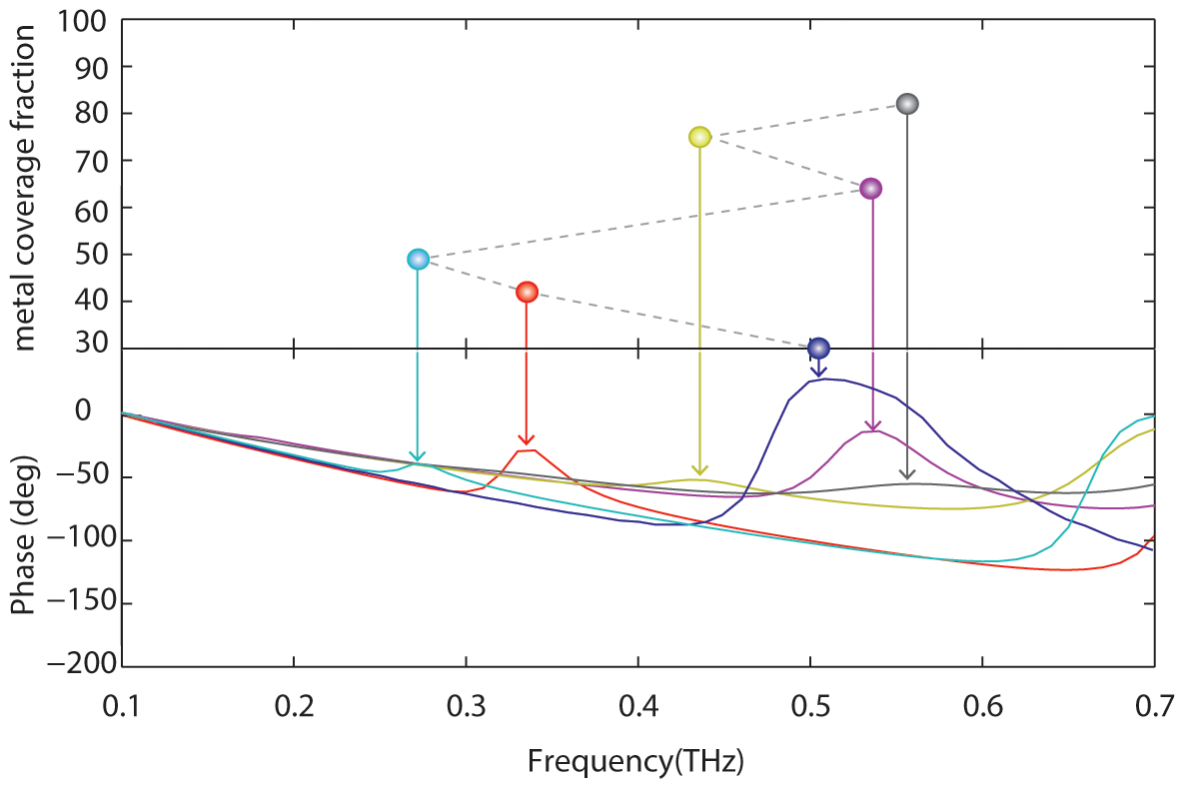
Phase of transmission for different metal coverage fraction in MSRs. The upper plot depicts the frequency at which maximum phase modulation takes place versus metal coverage fraction. The lower plot shows phase versus frequency for different metal coverage fractions.

As depicted in Table 1, it was observed that for MSRRs, the resonance always red-shifts as the metal coverage-fraction is increased. However, for MSRs, when the coverage-fraction is increased, the resonance frequency first starts red-shifting and then blue-shifts. Moreover, if the metal coverage-fraction is further increased (as depicted in Fig. 4), the response becomes even less monotonic. The first blue-shift is observed when the metal coverage-fraction is increased to larger values (i.e. from 50% to 64%). The trends observed in both structures can be qualitatively explained with an equivalent circuit model (the series of an equivalent inductance and an equivalent capacitance), see Refs. 16–17. Although, for the sake of simplicity, a two-arm MSR will be analyzed in the following discussion as an example to illustrate how the resonance frequency evolves when changing the metal coverage-fraction, analogous, i.e. non-monotonic trends, will also hold for four-arm MSRs.

**Figure 5:**
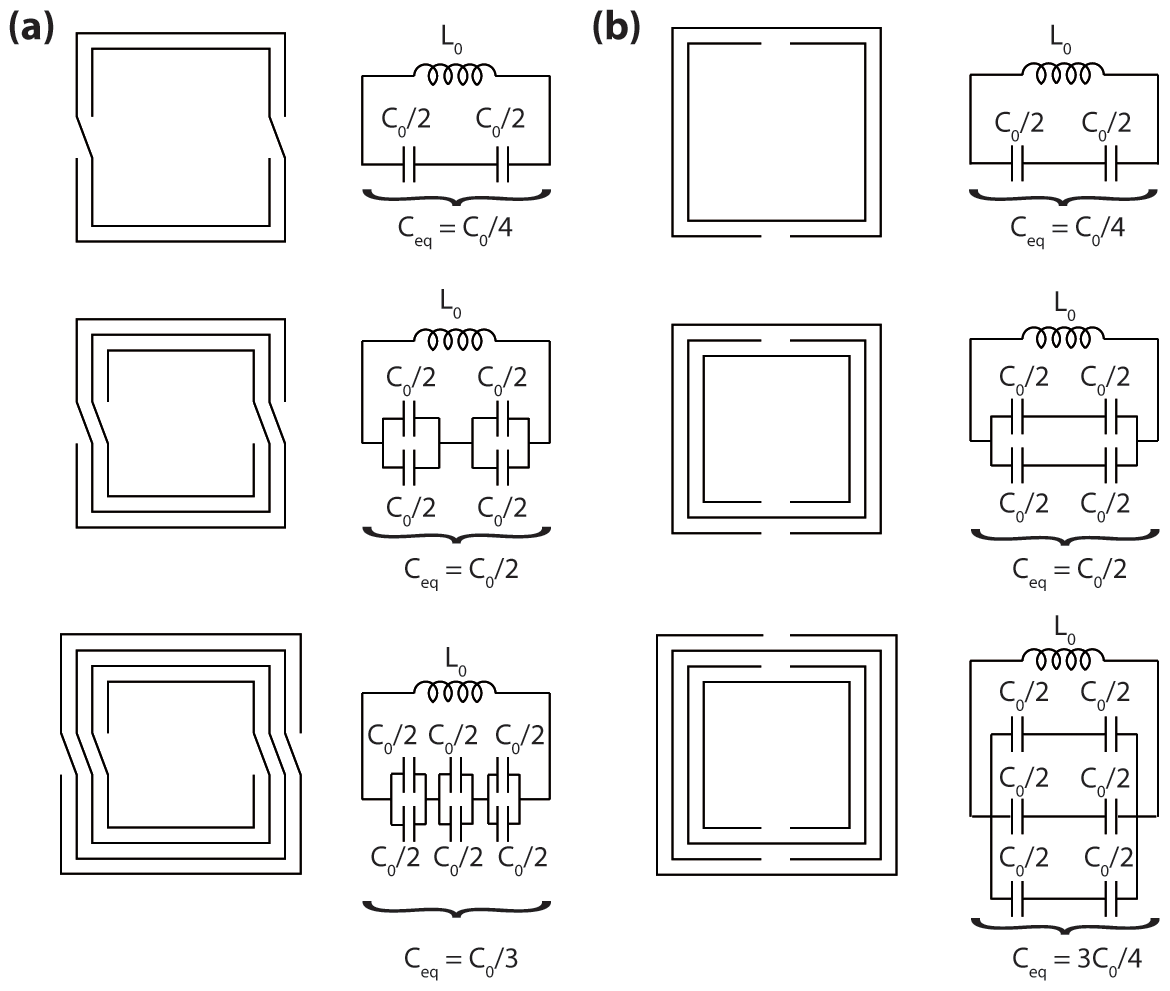
Equivalent circuit models. Different (a) MSR and (b) MSRR structures and their equivalent circuit model (following the discussion in Ref. 17). For MSRs equivalent capacitance first (at small number of turns) increases and then (at large number of turns) decreases when the number of turns is increased, whereas for MSRRs the equivalent capacitance always increases. Here C_0_ is the total capacitance between two adjacent rings, which is assumed to be the same for all the depicted structures.

For instance, for the case of MSRRs, the equivalent inductance (L_0_) is given by the average inductance of the rings. Therefore, the unit-cell size will be determinant in L_0_; since for the simulated structures the unit-cell dimensions remain almost constant, the equivalent inductance can be considered as independent of the metal coverage-fraction. Shown in Fig. 5(a)–(b) are sketches of the equivalent circuit models for MSR and MSRR geometries, respectively. These equivalent circuit might explain the behavior of these metamaterial with the change in metal coverage ratio. As previously discussed, for simplicity and for illustrative purposes here we discuss the case of a two-arm spiral structure (Fig. 5(a)), however the behavior of a four-arm spiral structure will follow analogous trends. Here C_0_ is the unity capacitance and L_0_ is the unity inductance (capacitance and inductance of two adjacent rings); in the figure it is assumed that this capacitance, which is related to the spacing between rings, remains constant as the spacing changes. The total inductance of these structure will not be largely affected when increasing the numbers of rings^16^,^17^, because the unit cell size is nearly constant and it can be approximated by the average ring size in the structure. It is worth mentioning that effects such as the capacitance between non-adjacent rings and the resistances arising from losses in the metal and the dielectric will be neglected. As the metal coverage-fraction increases (by adding more turns to the structure) the total effective capacitance of the structure increases as depicted in Fig. 5(b). In practice, because of the smaller spacing when coverage-fraction is increased, C_0_ also increases. This increase in effective total capacitance explains the observed red-shifting of the resonance frequency in MSRRs as the metal coverage-fraction is increased. As the metal coverage-fraction is increased, there are two competing effects taking place in this geometry: (i) as the number of turns increases, as depicted in Fig. 4(a), the equivalent capacitance first (at small number of turns) increases and then (at large number of turns) decreases. From this point of view, since indeed the number of turns is increased when the coverage-fraction is increased, the resonance is expected to blue-shift for very large metal coverage-fractions. In the other hand, (ii) as the coverage-fraction is increased, C_0_ increases due to a smaller spacing. From this point of view, the resonance is expected to red-shift. These two effects, (i) and (ii), are competing and both are important when increasing the coverage-fraction. For small coverage-fractions the same resonance-frequency evolution trend as in the MSRR is observed. However, for large coverage-fractions (i) can become dominant, and the overall effect we observe might be a decrease in the equivalent circuit capacitance, and therefore a resonance frequency blue-shift. However, when the metal coverage fraction is increased even further, (ii) can become dominant again and cause the resonance frequency to again red-shift; this leads to a very non-monotonic characteristic at large coverage-fractions.

It can be also observed (Fig. 4) that when the resonance red-shifts its strength diminishes, until eventually it becomes so weak that it disappears (blue, red, and sky-blue data points which correspond to 30%, 42%, and 49% metal coverage-fractions, respectively). When the metal coverage-fraction is further increased then a former higher-order resonance becomes the first resonance (e.g. purple data point, which corresponds to 64% metal coverage-fraction), leading to a saw-tooth characteristic for resonance-frequency versus metal coverage-fraction as observed in Fig. 4. The resonance strengths for metal coverage-fractions above 80% become considerably weaker since the gaps between adjacent gold-stripes become much smaller than the width of the gold-stripes and thus the structure, effectively, is mostly covered with metal.

**Figure 6:**
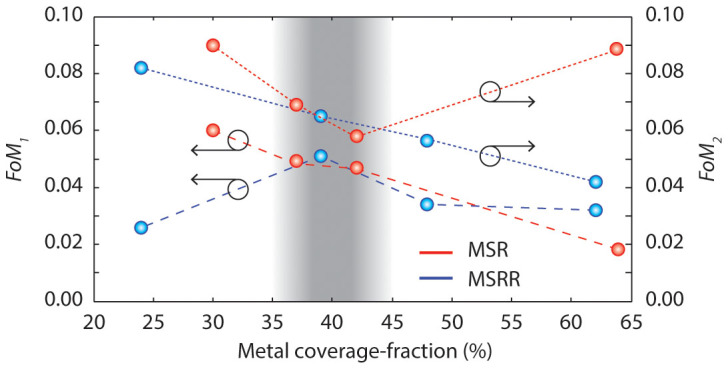
Figures of merit (*FoM_1_* and *FoM_2_*) versus metal coverage-fraction for both analyzed metamaterial geometries. In both cases, the best tradeoff occurs when the metal coverage-fraction is around 40% (gray shaded region).

Shown in Fig. 6 are the plots of *FoM_1_* and *FoM_2_* versus metal coverage-fraction for the two metamaterial geometries that were studied. When analyzing *FoM_1_*, it is observed that in MSRs the smallest coverage-fractions give the best tradeoffs. However, in MSRRs, decreasing the coverage-fraction below 30% significantly decreases transmission and phase modulation due to a very weak interaction between rings. Therefore, it can be noticed that moderate coverage-fractions (i.e. around 40%) give the best tradeoff.

When analyzing *FoM_2_*, in MSRRs, it can be observed that large coverage-fractions give the best tradeoff. This is a result of the monotonically increasing dependence of effective capacitance with coverage-fraction in this geometry. However, in contrast, for MSRs a completely different trend is observed when the metal coverage-fraction is increased. There is an optimal coverage-fraction, which occurs around 40%, that gives the best tradeoff. This is a result of the non-monotonic dependence of equivalent capacitance with coverage-fraction in this geometry.

When (overall) considering all the above described trends, it can be concluded that for both types of geometries the best tradeoff between *FoM_1_* and *FoM_2_* occurs in the region where coverage-fraction is around 40%. *From this point of view, deeply-scaled metamaterials can indeed provide a better performance than traditional metamaterials in beam-shaping applications. However, counterintuitively, use of very highly packed structures can actually be not beneficial. There is an optimal metal coverage-fraction, ~40%, which offers the best tradeoff. Interestingly, this optimal coverage-fraction is the same for both types of metamaterial geometries, MSRRs and MSRs.*

## Methods

### Numerical simulations and structural parameters

In the analyzed metamaterials, gold was chosen as the material for the metallic layers, whereas Al_2_O_3_ was considered as the dielectric-in-between (see Fig. 1). These materials were set in top of a 2-μm thick polyimide layer, which has the role of a substrate; the thickness of the gold and Al_2_O_3_ layers was 1-μm. The metamaterials were numerically simulated employing high frequency structural simulator (HFSS). In these simulations graphene was taken as a finite-thickness material with a 1-nm thickness, as discussed in Refs. 11, 18.

## Author Contributions

S.A. and B.S.-R. carried out the numerical simulations, analyzed the data, and contributed to the preparation of the manuscript.

## Figures and Tables

**Table 1:** Simulation results for the geometries depicted in Fig. 3

MSRR					MSR				
Metal coverage fraction	*f_p_* (GHz)	T @ *f_p_*	PM @ *f_p_*	unit cell (μm)	Metal coverage fraction	*f_p_* (GHz)	T @ *f_p_*	PM @ *f_p_*	unit cell (μm)
62%	220	60%	19°	(58)	64%	510	20%	33°	(52)
48%	290	34%	36°	(58)	42%	320	40%	42°	(54)
39%	350	28%	66°	(56)	37%	400	26%	67°	(52)
24%	440	17%	55°	(56)	30%	500	20%	108°	(54)

## References

[b1] Tonouchi M. Cutting-edge terahertz technology. Nature Photon. 1, 97–105 (2007).

[b2] Chen H. T. *et al.* Active terahertz metamaterial devices. Nature 444, 597–600 (2006). 17136089 10.1038/nature05343

[b3] Chen H. T. *et al.* A metamaterial solid-state terahertz phase modulator. Nature Photon. 3, 141–151 (2009).

[b4] Yu N. *et al.* Light Propagation with Phase Discontinuities: Generalized Laws of Reflection and Refraction. Science 334, 333–337 (2011). 21885733 10.1126/science.1210713

[b5] Kafesaki M., Shen N. H., Tzortzakis S. & Soukoulis C. M. Optically switchable and tunable terahertz metamaterials through photoconductivity. Journal of Optics 14, 114008 (2012).

[b6] Sensale-Rodriguez B. *et al.* Broadband graphene terahertz modulators enabled by intraband transitions. Nature Comm. 3, 780 (2012). 10.1038/ncomms1787 22510685

[b7] Sensale-Rodriguez B., Yan R., Liu L., Jena D. & Xing H. G. Graphene for reconfigurable terahertz optoelectronics. Proc. IEEE 101, 1705–1716 (2013).

[b8] Arezoomandan S., Yang K. & Sensale-Rodriguez B. Graphene-based electrically reconfigurable deep-subwavelength metamaterials for active control of THz light propagation. Applied Physics A 117, 423–426 (2014).

[b9] Lee S. H. *et al.* Switching terahertz waves with gate-controlled active graphene metamaterials. Nature Mater. 11, 936–941 (2012). 23023552 10.1038/nmat3433

[b10] Amin M., Farhat M. & Bagci H. A dynamically reconfigurable Fano metamaterial through graphene tuning for switching and sensing applications. Sci. Rep. 3, 2105 (2013). 23811780 10.1038/srep02105 PMC3696901

[b11] Yan R., Sensale-Rodriguez B., Liu L., Jena D. & Xing H. G. A new class of electrically tunable metamaterial terahertz modulators. Opt. Express 20, 28664 (2012). 23263104 10.1364/OE.20.028664

[b12] Gao W. *et al.* High-Contrast Terahertz Wave Modulation by Gated Graphene Enhanced by Extraordinary Transmission through Ring Apertures. Nano Lett. 14, 1242–1248 (2014). 24490772 10.1021/nl4041274

[b13] Carrasco E., Tamagnone M. & Perruisseau-Carrier J. Tunable graphene reflective cells for THz reflectarrays and generalized law of reflection. Appl. Phys. Lett. 102, 104103 (2013).

[b14] Liu M., Yin X. & Zhang X. Double-layer graphene optical modulator. Nano Lett. 12, 1482–1485 (2012). 22332750 10.1021/nl204202k

[b15] Ju L. *et al.* Graphene plasmonics for tunable terahertz metamaterials. Nat. Nanotechnol. 6, 630–634 (2011). 21892164 10.1038/nnano.2011.146

[b16] Bilotti F., Toscano A. & Vegni L. Design of spiral and multiple split-ring resonators for the realization of miniaturized metamaterial samples. IEEE Trans. Antennas. Propag. 55, 2258–2267 (2007).

[b17] Baena J. D. *et al.* Equivalent-circuit models for split-ring resonators and complementary split-ring resonators coupled to planar transmission lines. IEEE Trans. Microwave Theory and Techniques 53, 1451–1461 (2005).

[b18] Yang K., Liu S., Arezoomandan S., Nahata A. & Sensale-Rodriguez B. Graphene-based tunable metamaterial terahertz filter. Appl. Phys. Lett. 105, 093105 (2014).

